# Minimally-Invasive Diaphragmatic Plication in Patients with Unilateral Diaphragmatic Paralysis

**DOI:** 10.3390/jcm12165301

**Published:** 2023-08-15

**Authors:** Morris Beshay, Mohamed Abdel Bary, Volkan Kösek, Thomas Vordemvenne, Fritz Mertzlufft, Jan Schulte am Esch

**Affiliations:** 1 Department of General Thoracic Surgery, University Hospital OWL, Campus Bielefeld-Bethel, 33617 Bielefeld, Germany; 2 Department of Cardiothoracic Surgery, South Valley University, Qena 83523, Egypt; dr_abdelbary@yahoo.com; 3 Department of Thoracic Surgery, Klinik am Park, Klinikum, 44536 Luenen, Germany; volkan.koesek@klinikum-westfalen.de; 4 Department of Accident and Trauma Surgery, University Hospital OWL, Campus Bielefeld-Bethel, 33617 Bielefeld, Germany; thomas.vordemvenne@evkb.de; 5 Forschungsverbund BioMedizin Bielefeld, OWL (FBMB e.V.), Maraweg 21, 33699 Bielefeld, Germany; fritz.mertzlufft@evkb.de; 6 Department of General and Visceral Surgery, University Hospital OWL, Campus Bielefeld-Bethel, 33617 Bielefeld, Germany; jan.schulteamesch@evkb.de

**Keywords:** diaphragmatic paralysis, eventration, diaphragmatic plication, diaphragmatic repair, VATS

## Abstract

***Background:*** Diaphragm eventration (DE) represents a frequent problem with consecutive major impacts on respiratory function and the quality of life of the patients. The role of diaphragmatic plication (DP) is still underestimated. The aim of the present study is to evaluate the efficacy of minimally-invasive surgical diaphragmatic plication for the management of unilateral diaphragmatic eventration, to the best of our knowledge, this is the largest series reported in the literature using a non-resectional technique. ***Methods:*** All patients with unilateral diaphragmatic paralysis admitted for diaphragmatic plication (DP) between January 2008 and December 2022 formed the cohort of this retrospective analysis. DP procedure was done to plicate the diaphragm without resection or replacement with synthetic materials. Patients were divided into two groups: Group I included patients who underwent DP through an open thoracotomy, and Group II included patients who underwent DP through video-assisted thoracoscopic surgery (VATS). Data from all patients were collected prospectively and subsequently analyzed retrospectively. Patients’ characteristics, lung function tests, radiological findings, type of surgical procedures, complications, and postoperative follow-up were compared. The primary outcome measure was the postoperative result (deeper position of the paralyzed diaphragm) and improvement of dyspnea. The secondary outcome was lung function values over a long-term follow-up. ***Results:*** The study included a total of 134 patients who underwent diaphragmatic plication during the study period. 94 (71.7%) were males, mean age of 64 (SD ± 14.0). Group I (thoracotomy group) consisted of 46 patients (35 male). Group II (VATS-group) consisted of 88 patients (69 male). The majority of patients demonstrated impaired lung functions (*n* = 126). The mean length of diaphragmatic displacement was 8 cm (SD ± 113.8 cm). The mean duration of the entire procedure, including placement of the epidural catheter (EDC), was longer in group I than in group II (*p* = 0.016). This was also observed for the mean length of the surgical procedure itself (*p* = 0.031). Most patients in group I had EDC (*n* = 38) (*p* = 0.001). Patients in group I required more medication for pain control (*p* = 0.022). A lower position of the diaphragm was achieved in all patients (*p* < 0.001). The length of hospital stay was 7 (SD ± 4.5) days in group I vs. 4.5 (SD ± 3.2) days in group II (*p* = 0.036). Minor complications occurred in 3% (*n* = 4) in group I vs. 2% (*n* = 3) in group II. No mortality was observed in any of the groups. Postoperative follow-up of patients at 6, 12, and 24 months showed a significant increase in forced vital capacity (FVC) up to 25% (SD ± 10%–35%) (*p* = 0.019), in forced expiratory volume in 1 s (FEV1) up to 20% (SD ± 12%–38%) in both groups (*p* = 0.026), also in the diffusion capacity of carbon monoxide (DLCO) up to 15% (SD ± 10%–20%) was noticed in both groups. Chronic pain symptoms were noted in 13% (*n* = 6) in group I vs. 2% (*n* = 2) in group II (*p* = 0.014). Except for one patient in group II, no recurrence of DE was observed. ***Conclusions:*** Diaphragm plication is an effective procedure to reduce debilitating dyspnea and improve lung function in patients suffering from diaphragm eventration. Minimally invasive diaphragmatic plication using VATS procedures is a safe and feasible procedure for the management of unilateral diaphragmatic paralysis. VATS-DP is superior to open procedure in terms of pain management and length of hospital stay, hence, accelerated recovery is more likely. Careful patient selection is crucial to achieving optimal outcomes. Prospective studies are needed to validate these results.

## 1. Introduction

Unilateral diaphragmatic eventration (UDE) is defined as a permanent elevation of the hemidiaphragm without active muscular movement and without defects in its continuity. It occurs due to dysfunction or paralysis of the phrenic nerve, usually unilateral, with slightly more rates on the left side [1]. Acquired UDE has an incidence of about 0.05%. It is not a rare condition, with a slightly higher incidence in males. It is usually discovered incidentally during a routine chest X-ray (CXR), but dyspnea and orthopnea are the most common findings in symptomatic cases, especially during exercise. Dyspnea is usually due to compression of the lung tissue on the affected side, which is noticeable during exercise due to the paradoxical movement of the paralyzed diaphragm [2,3,4]. In patients with eventration or paralysis, diaphragmatic movement can be diminished, absent, or even paradoxical. As a result, ventilation and perfusion to the basal portion of the lung ipsilateral to the paralyzed or eventrated diaphragm are impaired, the latter possibly caused by regional vasoconstriction induced by alveolar hypoxiaIn advanced cases, dyspnea is aggravated by blood gas mismatch with increased CO_2_ due to partial atelectasis of the affected lung [5].

Congenital DE is a very rare condition and is associated with acute respiratory failure in the first few days after birth, especially when both sides of the diaphragm are affected. Most patients die within the first year of diagnosis. Acquired DE is usually diagnosed later, usually after the age of forty. It can result from a number of abnormalities that affect the neuromuscular axis between the cervical spinal cord and the diaphragm. The most common causes are idiopathic trauma, infection, cervical spondylosis, malignancy, thoracic or cardiac surgery, cryoablation intervention, and neuromuscular disorders [2,3,6].

The management of UDE depends on the etiology. Idiopathic, traumatic, and iatrogenic phrenic nerve palsy is the focus of this study and is the most common form in the vast majority of patients. In asymptomatic cases, no treatment is necessary. However, in symptomatic patients, UDE should be treated by surgical plication [7,8]. In the case of iatrogenic UDE due to nerve injury, patients should be observed for one to two years, as the function of the affected nerve may improve over time. However, in severe cases, observation should be limited to six months, and if no improvement occurs, surgery should be performed [9].

Surgical diaphragmatic plication (DP) has become popular in pediatric patients with respiratory failure but is still not frequently performed in adults [10,11]. Wright and colleagues demonstrated a major symptomatic improvement following diaphragmatic plication in a small group of select patients with significant improvement in objective measures of respiratory function. The procedure carried little attendant morbidity and no mortality [12].

The aim of DP is to return the flaccid, everted, and reluctating diaphragm to its normal position. Improvement of symptoms is achieved through various mechanisms, such as re-expansion of the lung, recruitment of more lung volume needed for gas exchange, and reduction of paradoxical diaphragmatic movement, which prevents the occurrence of blood gas mismatch. Demos demonstrated in a small series of patients the efficacy and durability of DP using a running suture [13,14]. Traditionally, this procedure is performed through a thoracotomy, which is a painful procedure, providing poor visibility of the elevated diaphragm and requiring longer anesthesia times. The minimally-invasive approach represents a valuable alternative to the open method and is becoming increasingly popular in the management of this condition [15]. The outcome after open DP and minimally invasive DP has not yet been compared. To the best of our knowledge, our study is the first and most comprehensive in the literature to compare open DP using thoracotomy and minimal invasive DP using the VATS approach.

## 2. Patients and Methods

### 2.1. Data Collection

Records of all 142 patients admitted to our center with a diagnosis of DE were analyzed over 15 years between January 2008 to December 2022. Eight patients were excluded from surgery due to other serious comorbidities or incomplete data. 134 patients were eligible for inclusion in the study. Data from these patients were collected from hospital medical records, general practitioners, pneumologists, and personal patient contact. Data from 134 patients were collected and retrospectively analyzed. All patients who underwent DP had a markedly elevated hemidiaphragm that persisted for at least 6 months prior to surgery, with significant dyspnea symptoms and restrictive lung function impairment. All patients were assessed for clinical dyspnea using the Modified Medical Research Council Dyspnoea Scale to assess the degree of baseline functional disability due to dyspnoea (mMRC) (Table 1). Patient’s characteristics as well as chest X-ray, sniff test, chest computed tomography as well as pulmonary function tests with the assessment of forced expiratory volume 1 (FEV1) and forced vital capacity (FVC) were collected. In addition, DLCO measurements were performed in most of the patients. For the measurements of the cupola displacement on the X-ray, a specific point was defined on the bony structure, namely the uppermost point of the upper edge of the first rib. Another point was set on the expected height of the cupola compared with the cupola of the healthy side. The difference between the actual height of the diaphragm and the expected height was taken as the distance of displacement of the cupola before surgery. The same measurements were performed after surgery to measure the distance of correction of the cupola. The preoperative values were compared with the postoperative values at 6, 12, and 24 months.

### 2.2. Surgical Procedure

**Figure 1:**
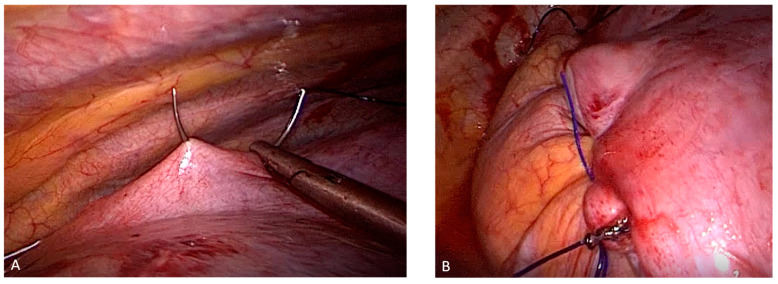
Plication of the diaphragm on itself (**A**: the first suture) after creating an inverted fold towards the abdominal cavity using nonabsorbable sutures (**B**: complete plication).

Diaphragmatic plication was performed by the same surgeons either by VATS procedure or via 8–10 cm lateral thoracotomy through the eighth intercostal space. VATS DP was performed under general anesthesia eighter using the standard 3 ports or uniportal (UVATS) approach. In all cases, a nasogastric tube was inserted to empty the stomach. The diaphragm with the abdominal contents was pushed caudally until the normal position of the cupola was reached. In this position, the reluctant cupola was folded with traction forceps. Plication was usually performed in two layers. The first was with interrupted PDS 0/0 U stitches by forming an inverted fold towards the abdominal cavity without resecting any tissue from the diaphragm. We usually started with the posterior part of the diaphragm towards the anterior part to avoid stomach wall involvement in the sutures (Figure 1). A second continuous suture is performed using polypropylene 0/0 as a safety suture. Care should be taken to avoid overcorrection of the diaphragm, especially on the left side, as it may lead to postoperative abdominal discomfort. After completing the repair, a chest tube was inserted, and the incisions were closed. For analgesia, a PDC was applied in the open group, and an intercostal nerve block with xylocaine was performed in VATS group. All patients were extubated directly in the operating theatre. A chest radiograph was taken postoperatively in all patients.

### 2.3. Statistical Analysis

Statistical analysis was performed using SPSS (SPSS version 22). Propensity score matching was used to adjust for differences in baseline characteristics. Descriptive statistics were presented for all patients and propensity-matched patients using a logistic regression model that included twelve variables: Age, sex, etiology, lung function tests, dyspnoea scale, ASA score, procedure (thoracotomy, VATS, UVATS, conversion), length of operative time, complications, length of hospital stay, reoperation, and recurrence. For time-to-operation analyses, comparisons for patients in group I and group II were made with Cox proportional hazards analysis. Results were expressed as mean ± SD for patients’ characteristics and other variables. Pearson’s correlation coefficient was used for correlation analysis, and a value of *p* < 0.05 was considered statistically significant.

## 3. Results

**Figure 2:**
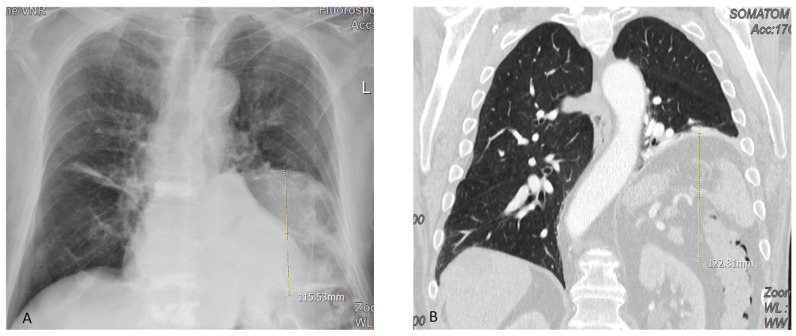
Right diaphragmatic eventration on the chest X-ray (**A**) and CT scan (**B**). The yellow line on (**A**) and (**B**) is an example of one patient’s actual length of diaphragmatic displacement.

Between January 2008 and December 2022, 142 patients with symptomatic diaphragmatic eventration (DE) were enrolled in the current study. Five patients were excluded from surgery due to serious comorbidity (advanced lung fibrosis: *n* = 3, advanced neurological diseases: *n* = 2). Three patients were excluded from the study due to incomplete data. All patients (*n* = 134) who underwent diaphragmatic plication during the study period were retrospectively analyzed. The cohort included 94 (70%) males, mean age was 64 (SD ± 14.0) years. CXR showed an elevated diaphragm on one side in all patients and on both sides in two patients (1.4%) with displacement up to 12 cm (Figure 2). The diagnosis of the elevated diaphragm was confirmed in all patients for more than 2 years in 29 patients (22%), between 1–2 years in 45 patients (33%), and <1 year in 60 (45%).

**Figure 3:**
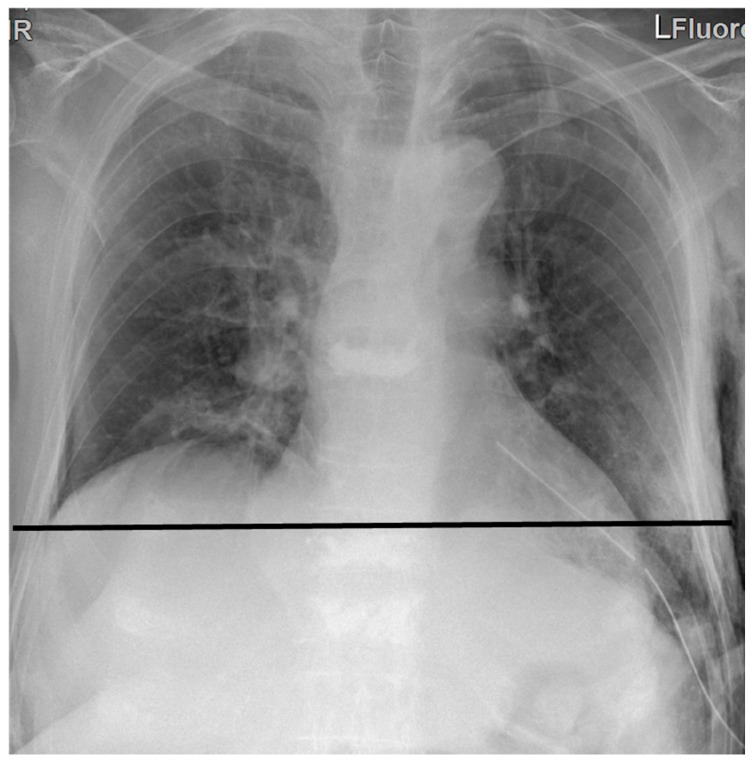
Postoperative chest X-ray after plication of the left side of the diaphragm. The black line demonstrates the new level after the plication of the left cupola.

**Figure 4:**
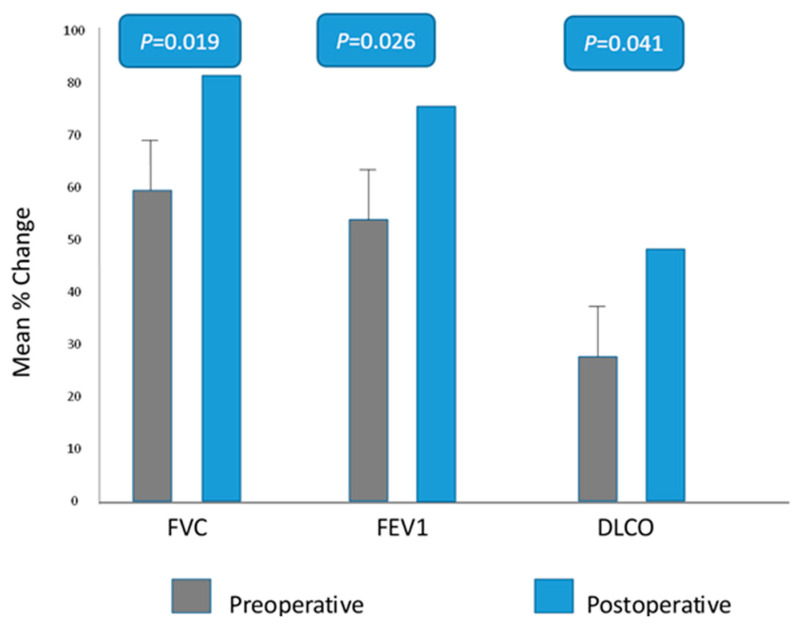
Lung functions and DLCO tests preoperative in grey and 12 months postoperative control in blue.

Preoperative characteristics are shown in Table 2. A fluoroscopy with sniff testing was performed in most of the cases 74% (*n* = 99). 127 patients had a paradoxical elevation of the diaphragm with sniff, and 10 patients had varying degrees of subjectively reduced diaphragmatic downward displacement with sniff. Patients were divided into two groups according to the type of surgery: Group I (thoracotomy group) consisted of 46 patients (35 males). Group II (VATS-group) consisted of 88 patients (69 males). Other patients’ characteristics are summarized in Table 3. All patients received DP without diaphragmatic resection or using any kind of prosthesis. Two patients in group II were converted from VATS to mini-thoracotomy due to massive adhesions. A deeper position of the diaphragm was achieved in all patients (*p* < 0.001) (Figure 3). Minor complications occurred in 3% (*n* = 4) in group I vs. 2% (*n* = 3) in group II. Other operative findings are shown in Table 4. No mortality was observed in any of the groups. Clinical follow-up of patients at 6, 12, and 24 months showed a significant increase in forced vital capacity (FVC) up to 25% (SD ± 10%–35%) (*p* = 0.019), in forced expiratory volume in 1 s (FEV1) up to 20% (SD ± 12%–38%) in both groups (*p* = 0.026), as well as in the diffusion capacity of carbon monoxide (DLCO) up to 15% (SD ± 10%–20%) in both groups (Figure 4). Arterial blood gas analysis showed a significant decrease in the CO_2_ values under exercise compared with preoperative values (*p* = 0.018). Six-minute walking distance (6-MWD) was significantly improved by 98% (*n* = 131) (*p* = 0.021). However, no statistical difference was noted between the two groups regarding lung function testing and 6-MWD. Patients in group I required more medication for pain control, especially after the removal of the epidural catheter (*p* = 0.022). No paradoxical movement of the diaphragm was observed postoperatively (*p* < 0.001). During a median follow-up period of up to 24 months, no recurrence was noted except for one patient in group II who developed a diaphragmatic hernia three months after VATS-DP. The reoperation revealed a diaphragmatic defect with protrusion of the great omentum, which was treated with repositioning and direct repair. Chronic pain symptoms were reported in 13% (*n* = 6) in group I vs. 2% (*n* = 2) in group II (*p* = 0.014). Other postoperative outcomes are shown in Table 5. Basline charachteritics before and after propensity score (PS) matching are showen in Table S1.

## 4. Discussion

**Figure 5:**
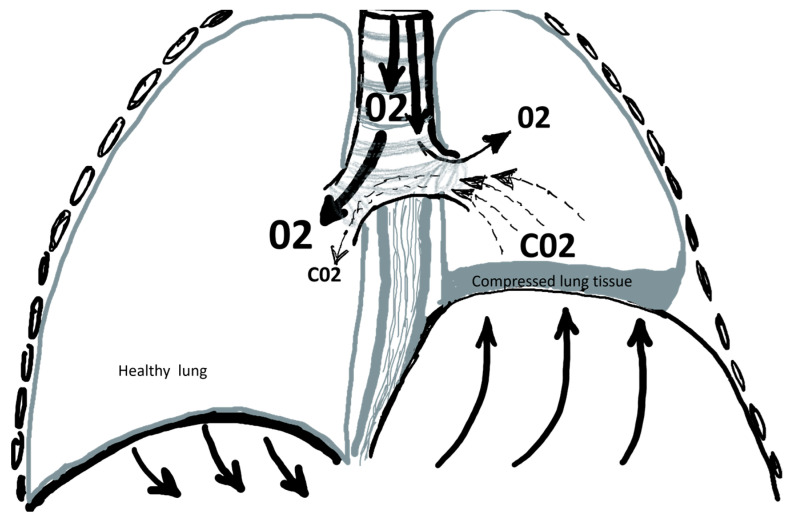
Demonstration of the pathophysiological effect of the paralyzed elevated diaphragm on the gas mismatch.

Diaphragmatic eventration (DE) is an elevation of the diaphragm above its usual height, accompanied by compression of the adjacent lung tissue. Although DE is well-known, many general practitioners and pneumologists are still not aware of the possible surgical management. This could be due to one or more of the following factors: There is often no association between symptoms and radiological findings, family doctors and pneumologists are reluctant to refer the patients, surgeons are reluctant to operate on patients, lack experience in performing the procedure, especially in minimally invasive technique, and finally the lack of awareness of surgical treatment of DP [9,10]. Many patients suffer from dyspnea for many years despite undergoing complex conservative treatment. Orthopnea and dyspnea are the most common symptoms and typically worsen with swimming, leaning forward, exercising, climbing stairs, or walking uphill [16,17]. Most patients in this study suffered from moderate to severe dyspnea with impairment in their daily activities. The pathophysiological explanations for these findings are still unclear. According to other reports, we postulate the following pathophysiological mechanisms in supine positions: *(1) The paradoxical movement of the affected diaphragm:* This happens because the intrathoracic negative pressure created during inspiration pushes the healthy diaphragm toward the abdominal cavity. This causes the paralyzed hemidiaphragm to rise in the cephalic direction [16,17,18,19,20]. *(2) Gas mismatch problems on the affected side:* The elevation of the paralyzed diaphragm compresses the lung, causing CO_2_ to be released upwards from the lung into the bronchial tree and the trachea. At the same moment, during the inspiration process, air containing O_2_ flows down through the trachea. At this moment, a shunt occurs at the lower part of the trachea, in the main bronchus, and in the bronchi of the affected side. Therefore not enough O_2_ can reach the alveoli of the affected lung. *(3) Gas mismatch problems on the healthy side:* A small amount of the compressed CO_2_ from the compressed lung can reach the healthy lung through the rapid airflow of the inhaled air, which again would lead to the mixing of the inhaled O_2_ with CO_2_ during the inhalation phase (Figure 5). *(4) Wasted work of breathing*: There is a loss of energy through the activation of other supportive respiratory muscles to compensate for the gas imbalance and symptoms of dyspnea [20,21]. Other clinical symptoms include chest pain, recurrent respiratory infections, dyspepsia, and palpitations caused by cardiac arrhythmias [3,22]. Various surgical techniques have been proposed for the treatment of diaphragmatic eventration, e.g., excision of the reluctating diaphragm and closure with a suture, excision using stapling devices, excision and replacement with synthetic materials, e.g., polypropylene mesh, etc. In this study, we adopted a plication technique without the resection of diaphragmatic tissue or the application of a prosthesis. Our experience has shown that a simple diaphragmatic plication is very effective and easy to perform, in contrast to other time-consuming methods [23,24]. Unilateral traumatic rupture of the diaphragm as a result of blunt or penetrating trauma is challenging to detect in the initial trauma setting. This is especially true when diaphragmatic trauma is part of a polytrauma. Complications of undetected diaphragmatic defects with incarcerating bowel are rare but can be serious. A thorough investigation should always be undertaken in cases of blunt abdominal and thoracic trauma to exclude diaphragmatic injury in order to avoid post-traumatic complications [25]. DP through a standard thoracotomy is still the most commonly performed technique. Very few surgeons would perform it using VATS techniques, which were first described by Mouroux and colleagues [26]. Our study showed that VATS-DP is superior to the open method in many aspects. The duration of the entire procedure was longer with the open surgery than with the VATS method, postoperative pain, and the length of hospital stay. Nevertheless, in the past decade, the minimally invasive technique has gained acceptance because it has low morbidity and mortality rates [22,23,24]. In contrast to Demos and colleagues, we used to do the DP using two layers of sutures: The first one is an interrupted suture. The role of this first suture is to estimate the ideal final level at which one wants to place the diaphragm and to create the optimal degree of tension for a plication. The second suture Is a running suture with a non-absorbable polypropylene suture that secures the first interrupted sutures. In this way, failure of one suture does not lead to the failure of the entire plication [12]. In contrast to other reports, where a fully thoracoscopic approach was associated with a high complication rate, our results showed a lower complication rate as well as a shorter duration of hospital stay. Consistent with other reports, our results showed longer operative times for VATS Procedures on the right side due to the difficulty of replacing the luxated abdominal organs downwards with VATS instruments in patients with very high diaphragm on the right side [27,28,29]. No significant difference was found between patients who underwent VATS-DP with the standard multi-portal technique and those who underwent UVATS in terms of pain control, duration of surgery, length of hospital stay, or occurrence of complications. In this study, we found that DE was slightly higher on the left side (57%), but laterality, as such, had no statistical differences in primary or secondary outcomes. Finally, it is important to be careful to avoid injury of any abdominal organs during suturing of the diaphragm. It is also important not to overcorrect, as this can lead to postoperative abdominal discomfort.

Although the diagnosis of DE is easy to make by clinical examination and chest radiography, the etiology of DE is difficult to find out but should be carefully investigated. Despite exhaustive investigations, the etiology may remain unclear [3]. In our opinion, cervicothoracic and upper abdominal CT scans should be performed in every patient to rule out both neoplastic disease (cervical, mediastinal, or pulmonary) involving the phrenic nerve and subdiaphragmatic abnormalities. In our study, most patients had idiopathic DE, but the second most common cause of DE was post-traumatic, especially after a history of a car accident followed by an iatrogenic lesion after thoracic or cardiac surgery. We recommend DP procedure in patients with prominent symptoms who have had evidence of diaphragmatic eventration for at least more than six months.

Many reports showed functional improvement even at long-term follow-ups, with significant improvement in respiratory functions [29,30]. Similarly, Higgs et al. followed 19 patients for 10 years and reported DP to be an appropriate choice for improving respiratory function, dyspnea score, and patient satisfaction [28]. Freeman et al. reported improvement in spirometry values in 41 case series at six months and long-term follow-up [30]. Ribet et al. found a 15% increase in FEV1 and a 20% increase in vital capacity after plication [28]. Calvinho et al. described the long-term follow-up of 20 patients with a maximum duration of up to 17 years. Most patients had significant improvement in their daily activities postoperatively [31]. The selection of suitable patients for DP is crucial for the outcome and patient satisfaction. During the study period, eight patients were excluded from surgery due to advanced morbidity or incomplete preoperative investigations. A few patients had advanced pulmonary fibrosis or uncompensated cardiomyopathy, and last but not least, there were advanced neurological disorders. Two patients had bilateral DE, both were also excluded from surgery. Extrem obesity could be considered a relative contraindication. Usually, we encourage the patients to lose weight before performing the DP. Most of the studies mentioned above were conducted in children or in patients with mechanical respiratory support. Our study is the largest study including a larger number of adult patients, which showed similar results with a 20% improvement in FEV1 and a 25% improvement in FVC with long-lasting effects. However, in contrast to Özkan et al., who reported an improvement in postoperative FEV1 at one month, but no clinically significant increase in lung functions in long-term outcome was observed [32], our results showed improvement of the FEV1 values at long-term follow-up.

## 5. Conclusions

We conclude that adult patients with chronic dyspnea due to UDE would significantly benefit from DP. DP showed significant improvement in patient functional status, which is closely correlated with a radiological and sonographic assessment. Minimally-invasive procedures using VATS techniques for plication of the diaphragm is a safe and effective procedure associated with prompt postoperative recovery, shorter operative times, less use of pain medications, and shorter hospital stay. VATS DP should replace the open procedure to avoid the hazards of thoracotomy. Prospective studies are needed to support our findings.

## Figures and Tables

**Table 1:** The Modified Medical Research Council (mMRC) Dyspnea Scale.

Description	Grade
I only get breathless with strenuous exercise	0
I get short of breath when hurrying on level ground or walking up a slight hill	1
On level ground, I walk slower than people of my age because of breathlessness, or I have to stop for breath when walking at my own pace on the level	2
I stop for breath after walking about 100 yards or after a few minutes on level ground	3
I am too breathless to leave the house or I am breathless when dressing/undressing	4

**Table 2:** Preoperative characteristics.

Characteristics	*n*	%	*P*
Cause:			
Idiopathic	99	74	0.039
Trauma:	15	13	
● blunt thoracic trauma	4	3	
● blunt cervical trauma	11	8	
Iatrogenic:	17	13	
● Cryoablation	9	7	
● Cardiac surgery	5	4	
● Thoracic surgery	3	2	
Cervical spondylosis	2	1	
● Lung functions:	126	94	
● Restrictive values	86	64	0.053
● Obstructive values	12	9	
● Combined values	40	35	
● No lung function done	8	6	
ABG-Analysis:	134	100	
● Good (O_2_ > 70 mmHg, CO_2_ < 45 mmHg)	102	76	
● Intermediate (O_2_ 65–70 mmHg, CO_2_ 45–50 mmHg)	18	13	
● Bad (O_2_ < 65 mmHg, CO_2_ > 50 mmHg)	14	10	
Sniff test results:	131	98	
● Paradoxical motion	127	94	0.004
● Reduced contractility	4	3	
● Eventration only	6	4	

**Table 3:** Other preoperative patient’s characteristics.

Patient Characteristics	Results	*P*
Age years (mean ± SD)	64 ± 14 years	
Gender		
-Male	104	0.021
-Female	30	
Race *n* (%)		
-Caucasian	128 (97%)	0.001
-Hispanic	6 (3%)	
Smoking status *n* (%)		
-Never smoker	14 (16%)	0.003
-Smoker/previous smoker	120 (85%)	
Side		
-Left	79 (59%)	0.54
-Right	55 (39%)	0.62
Comorbidities		
-Diabetes	15 (11%)	0.41
-COPD	48 (36%)	0.62
-Hypertension	32 (24%)	0.52
-Corornary artery disease	27 (20%)	0.57
ASA Classification		
-ASA 1	88	0.03
-ASA 2	32	0.01
-ASA 3	1	0.002
Height of diaphragm om the X-ray (cm)	8 ± 4	0.018
Dyspnea scale (MRC) Grade *n* (%)		
	2 8 (7.5%)	
	3 109 (70%)	
	4 17 (22.5%)	

**Table 4:** Operative findings.

Characteristics	*n*	%	*P*
Length of the whole procedure (minutes)			0.016
Group I	135.2 (SD ±48.5)		
Group II	98.2 (SD ± 36.2)		
Length of the operative time (minutes)			0.031
Group I	62.2 (SD ± 41.6)		
Group II	51.2 (SD ± 32.4)		
Epidural Catheter	40	30	
Group I	38		0.001
Group II	2		
Type of incision:			
Lateral thoracotomy	42	31	
Posterolateral thoracotomy	4	3	
3 ports VATS	81	60	
UVATS	7	5	
Conversion from VATS to thoracotomy	2	1.4	
Type of sutures			0.001
One layer U-Sutures	1 0.7		
One layer continuos suture	9	7	
Two layers (interrupted and running) 124 92	124	92	
Intraoperative complications			
Bleeding	1	0.7	
Lung injury	1 0.7		
Injury to abdominal structures	0	0	
Blood transfusion	0	0	
Duration of chest tube			0.062
Group I	3		
Group II	2		

**Table 5:** Postoperative findings.

Characteristics	*n*	%	*P*
Position of the plicated diaphragm on X-ray			0.001
Excellent (diaphragm reaches its original position)	125	93	
Satisfactory (2–3 cm higher than the original position)	9	7	
Complications	7	5	0.021
Abdominal			
Discomfort	4	3	
Vomiting	1	0.7	
Obstipation	3	2	
Respiratory			
Atelectasis	0	0	
Pneumonia	1	0.7	
Air leakage	2	1.4	
Wound infection			
Superficial	1	0.7	
Deep	0	0	
Length of hospital time	5 (SD ± 3.2)		0.036
Group I	7 (SD ± 4.5)		
Group II	4.5 (SD ± 3.2)		
Symptoms at discharge			0.003
Group I	2	1.4	
Group II	0 0		
Chronic Pain			0.014
Group I 6	4		
Group II 2	1.4		

## Data Availability

Tha data of all patients are availble on request.

## Supplementary Materials

The following supporting information can be downloaded at: https://www.mdpi.com/article/10.3390/jcm12165301/s1, Table S1: Baseline characteristics before and after propensity Score (PS) Matching.

## Abbreviations

DE	diaphragmatic eventration
DP	Diaphragmatic plication
UDP	unilateral diaphragmatic paralysis
VATS	video-assisted thoracic surgery
UVATS	uni portal video-assisted thoracic surgery
EDC	epidural catheter
FVC	forced vital capacity
FEV1	forced expiratory volume in 1 s
DLCO	diffusion capacity of carbon monoxide
UDE	Unilateral diaphragmatic eventration
CXR	chest X-ray
US	ultrasound
COPD	chronic obstructive lung disease
ASA	American Society of Anesthesiologists
MRC	Medical Research Council
