# Supplementary material for: Minimally-Invasive Diaphragmatic Plication in Patients with Unilateral Diaphragmatic Paralysis
Source: J Clin Med. 2023 Aug 15;12(16):5301. doi: 10.3390/jcm12165301 (PMC10455218; doi:10.3390/jcm12165301)
Supplement: Supplementary file 1 [file jcm-12-05301-s001.zip › jcm-2498950-supplementary.pdf]

Table S1. Baseline characteristics before and after propensity Score (PS) Matching

Characteristic	Before Matching Group I (n=46)	Before Matching Group II (n=88)	Before Matching *P*	After Matching Group I (n=46)	After Matching Group II (n=46)	After Matching *P*
Age, Years (mean±SD)	56±11	62±8	0.63	58±9	61±12	0.66
Male	35	69	0.021	35	37	0.81
Caucasian	41	87	0.001	42	41	0.9
Smoker/previous smoker	39	81	0.004	39	43	0.073
Affected side (left)	28	51	0.048	28	42	0.054
Diabetes	5	10	0.041	5	10	0.041
COPD	15	33	0.055	15	28	0.062
Hypertension	13	19	0.083	13	21	0.052
Corornary artery disease	11	16	0.069	11	19	0.057
ASA 1	36	52	0.048	25	45	0.03
ASA 2	10	22	0.053	4	28	0.01
ASA 3	2	5	0.07	0	1	0.002
Height of diaphragm on the X-ray (cm)	9±16	7±12	0. 61	2±1	1±3	<0. 018
Paradoxical movement/undefined	43	84	0.071	1	0	0.004
Dyspnoea scale (MRC) Grade 2	3	5	0.08	4	2	0.062
Dyspnoea scale (MRC) Grade 3	34	76	0.059	3	5	0.01
Dyspnoea scale (MRC) Grade 4	6	11	0.06	1	2	0.038
Lung functions; Restrictive values	29	57	0.053	3	9	<0.001
Lung functions; obstructive values	4	8	0.031	4	8	0.031
Lung functions; combined values	15	25	0.052	11	18	0.054
FVC % (SD±)	55±12	58±15	0.067	77±12	88±6	0.019
FEV1 % (SD±)	53±17	54±18	0.062	68±6	78±13	0.026
DLCO % (SD±)	36±10	39±18	0.058	41±3	48±2	0.041
Hospital stay, Days	7	4.5	0.036	7	4	0.033
Complications	4	3	0.7	4	1	0.021
Recurrence	0	0		0	0	
